# Colon cancer underwent surgical resection after chemotherapy combined with immunotherapy: Case report

**DOI:** 10.1097/MD.0000000000042575

**Published:** 2025-05-30

**Authors:** Zhibin Ye, Jingpeng Li, Yuqin Zang, Fan Yang, Wei Geng

**Affiliations:** a Department of Gastrointestinal Surgery, Hebei General Hospital, Shijiazhuang, PR China; b Graduate School, Hebei Medical University, Shijiazhuang, PR China; c Department of Oncology, Hebei General Hospital, Shijiazhuang, PR China; d Department of Pathology, Hebei General Hospital, Shijiazhuang, PR China.

**Keywords:** colon cancer, immunotherapy, neoadjuvant therapy, pathological complete response

## Abstract

**Rationale:**

Neoadjuvant immunotherapy combined with chemotherapy can offer a higher surgical conversion rate for patients with locally advanced unresectable colon cancer while maintaining safety.

**Patient concerns:**

Patients are primarily concerned about treatment plans and their efficacy, including the number of cycles for neoadjuvant therapy, the timing of surgery, and potential side effects associated with the treatment.

**Diagnoses:**

Colonoscopy and biopsy were conducted to confirm the malignant nature of the colon tumor. A computed tomography scan of the abdomen showed that the maximum diameter of the tumor was 6.5 cm×5.5 cm, and the tumor markedly invaded adjacent tissues, making it inoperable.

**Interventions:**

After 3 cycles of neoadjuvant therapy, a reexamination of abdominal computed tomography showed that the patient reached the criteria for surgery. Subsequently, a laparoscopic radical right hemicolectomy was conducted.

**Outcomes:**

The surgery was successful and no surviving tumor cells were seen in postoperative pathological examination.

**Lessons:**

Surgical resection of colon cancer after chemotherapy combined with immunotherapy can achieve R0 resection and pathological complete response, which is a complex but promising treatment.

## 1. Introduction

The exact pathogenesis of colon cancer remains unknown, but it may be the result of a combination of internal and external factors. Most colon cancers are adenomatous, with adenomatous polyps, inflammatory bowel disease, and family history serving as risk factors. In addition, excessive dietary intake of fatty proteins, lack of dietary fiber, age, obesity, race, and smoking increase the risk of colon cancer. Colon cancer remains asymptomatic in the early stages, and some patients present with localized progression, making treatment challenging. With the continuous improvement of our center, we recommend our patients receive neoadjuvant chemotherapy combined with immunotherapy before radical surgery to improve the survival rate. Here, we report a patient undergoing surgical resection after neoadjuvant chemotherapy combined with immunotherapy who achieved R0 resection and pathological complete response. This case demonstrated the feasibility and effectiveness of chemotherapy combined with immunotherapy and provided hope and confidence for more patients. The clinical significance lies in the application of chemotherapy combined with immunotherapy, which makes the treatment of colon cancer more individualized. Detection of tumor molecular markers (e.g., PD-1, MMR status, RAS/BRAF gene mutations, etc) can guide the development of treatment plans and select the most suitable therapeutic plan for patients, thus improving the treatment response.^[1]^

## 2. Case report

**Figure 1.**
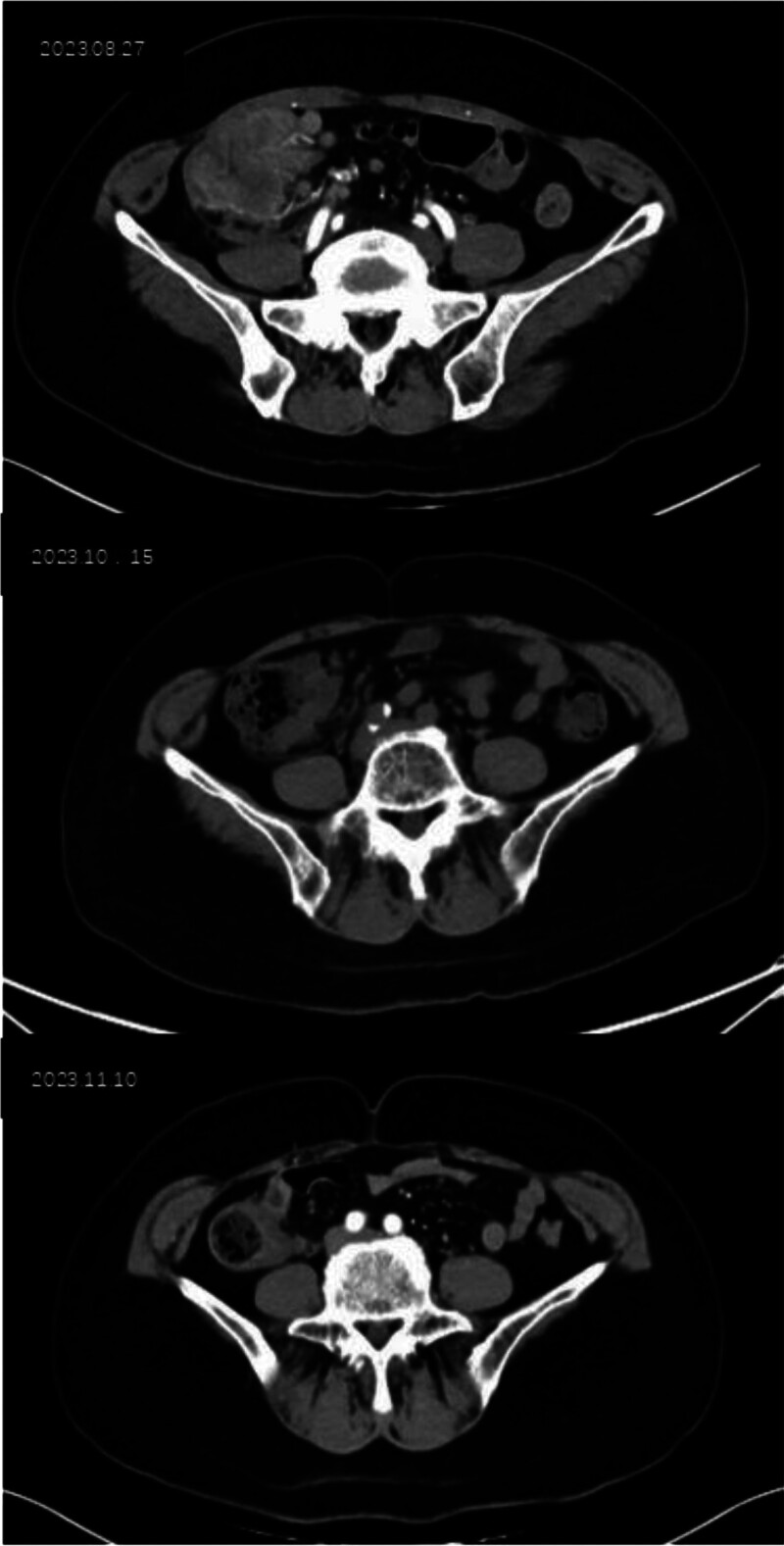
CT scans of the abdomen show changes in maximum tumor diameter and the mesenteric lymph nodes over time. CT = computed tomography.

**Figure 2.**
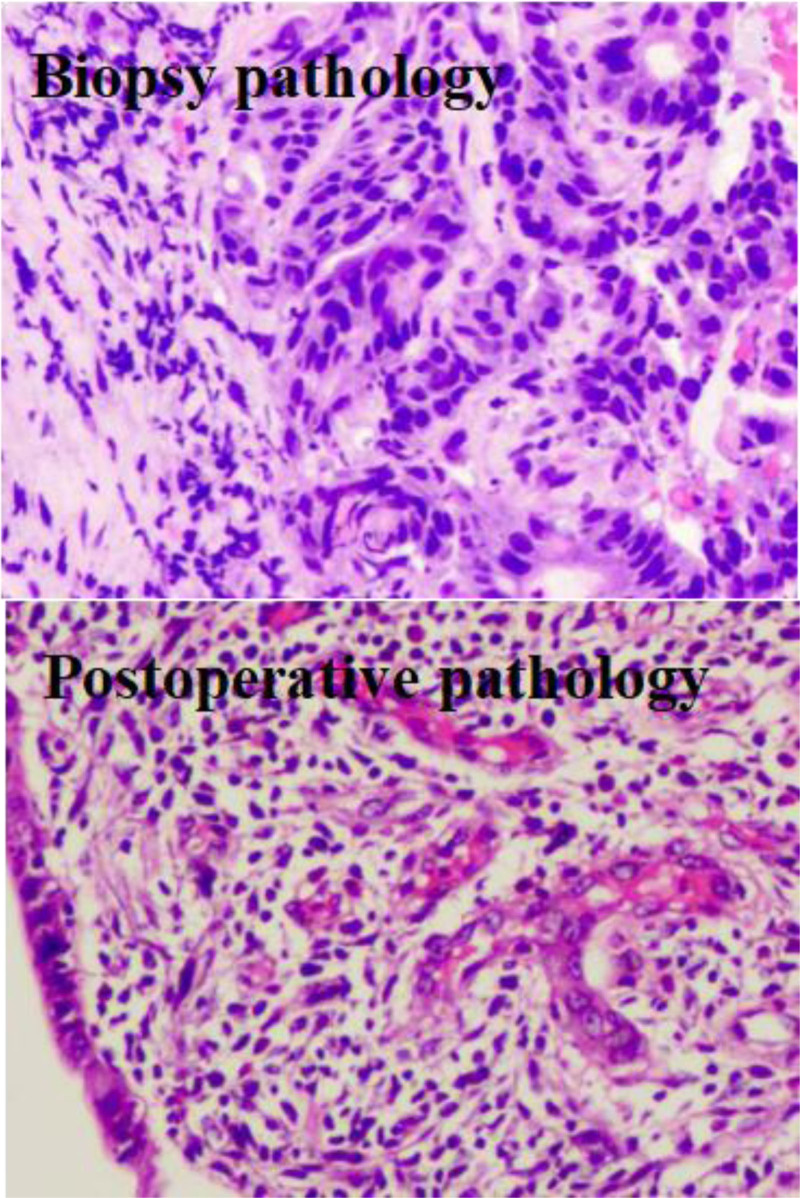
Biopsy pathological images were compared with postoperative pathological images (hematoxylin–eosin stain 400).

A 60-year-old female patient suffering from right abdominal pain for twenty days was admitted to our hospital on August 27, 2023. On physical examination, there was a mass in the right lower abdomen. Colonoscopy and biopsy were conducted to confirm the malignant nature of the colon tumor. Computed tomography scan of the abdomen showed that the maximum diameter of the tumor was 6.5 × 5.5 cm, and the tumor markedly invaded adjacent tissues, making it inoperable. Gene detection in the biopsy of the patient suggested that microsatellite instability-high (MSI-H). According to the 2024 Chinese Society of Clinical Oncology guidelines for colorectal cancer, immunotherapy can be used in conversion therapy for MSI-H patients, but it is not the preferred 1st-line option. And after fully considering the patient’s treatment preferences, a combined approach of chemotherapy and immunotherapy was selected (scheme: oxaliplatin 130 mg/m^2^ plus capecitabine 1000 mg/m^2^ plus sindilizumab 200 mg, Q21). Reexamination after 2 cycles revealed that the tumor size and the number of metastatic lymph nodes were both reduced. After 3 treatment cycles, the tumor size and the number of metastatic lymph nodes were also significantly reduced (Fig. 1), and no severe toxic or side effects were observed during treatment. Laparoscopic radical right hemicolectomy was conducted under general anesthesia on November 14, 2023. The surgery was successful. Postoperative pathology revealed that all parts of the original tumor bed were resected. In addition, severe chronic inflammation of the mucosa, multifocal foam cell aggregation, lymphocyte infiltration, calcification, and foreign body giant cell reaction were observed in the whole intestinal wall, and no surviving tumor cells were observed in pathological assessment. In pathological assessment after chemotherapy, there were no viable tumor cells (tumor regression grade: 0, complete response), intravascular cancer thrombi, nerve infiltration (Fig. 2), and regional lymphatic metastasis. Ten days after surgery, the patient was discharged from the hospital after eating and defecating without any abnormality.

## 3. Discussion

The application of immunotherapy in the neoadjuvant and adjuvant treatment of colon cancer has yielded promising results, especially in patients with high MSI-H or defective mismatch repair, who weakly respond to conventional treatments but exhibit satisfactory responses to immunotherapy in the early stages of the disease.^[2,3]^ Immunotherapy may need multiple cycles to achieve optimal results. However, there is no standardized number of cycles so far, and the number of cycles should be determined based on the patient’s specific situation and the physician’s assessment. For locally advanced colon cancer, the choice of treatment has become a headache. Immunotherapy combined with chemoradiation can lead to better surgical regression rates and complete pathological remission but is associated with more side effects and postoperative complications.^[4]^ Immunotherapy may enhance the antitumor response of the immune system, which may simultaneously damage normal tissues and organs and lead to inflammatory bowel disease and hyperthyroidism.^[5]^ Overcoming primary and secondary resistance and recognizing pseudoprogression and over-progression of neoadjuvant immunotherapy are difficult clinical issues that need attention.^[6,7]^ In the event of pseudoprogression, several aspects such as the patient’s clinical stability, tumor marker levels, and imaging changes, are needed to continue immunotherapy.^[8–12]^ Assessment of the tumor immune microenvironment and peripheral immunological features are crucial for colorectal cancers with microsatellite stable or proficient mismatch repair. In addition, searching for effective efficacy-predicting biomarkers is an important research direction. In the present case, we did not measure PD-1/PDL1 before treatment, which may be a pitfall in our treatment plan since adequate evaluation should be considered before treatment.

## 4. Conclusion

Surgical resection of colon cancer after chemotherapy combined with immunotherapy can achieve R0 resection and pathological complete response, which is a complex but promising treatment. Selection of a reasonable treatment plan and formulation of an individualized treatment strategy can optimize the therapeutic effect and survival rate of patients. At the same time, with the continuous progress of medical technology and the emergence of new treatment methods, more patients will benefit from this comprehensive treatment in the future.

## Acknowledgments

We acknowledge the contributions of the colleagues in Hebei General Hospital that aided the efforts of the authors.

## Author contributions

**Conceptualization:** Zhibin Ye.

**Investigation:** Zhibin Ye, Fan Yang.

**Methodology:** Yuqin Zang, Wei Geng.

**Writing – original draft:** Zhibin Ye.

**Writing – review & editing:** Zhibin Ye, Jingpeng Li.

## References

[1] Chalabi M Verschoor YL van den Berg J. LBA7 Neoadjuvant immune checkpoint inhibition in locally advanced MMR-deficient colon cancer: the NICHE-2 study. Ann Oncol. 2022;33(Suppl 7):S1389–S869.

[2] Cercek A Dos Santos Fernandes G Roxburgh CS. Mismatch repair-deficient rectal cancer and resistance to neoadjuvant chemotherapy. Clin Cancer Res. 2020;26:3271–9. 32144135 10.1158/1078-0432.CCR-19-3728 PMC7348681

[3] Andre T Shiu K-K Kim TW. Pembrolizumab in microsatellite-instability-high advanced colorectal cancer. N Engl J Med. 2020;383:2207–18. 33264544 10.1056/NEJMoa2017699

[4] Chalabi M Fanchi LF Dijkstra KK. Neoadjuvant immunotherapy leads to pathological responses in MMR- proficient and MMR-deficient early-stage colon cancers. Nat Med. 2020;26:566–76. 32251400 10.1038/s41591-020-0805-8

[5] Haanen J Carbonnel F Robert C. Management of toxicities from immunotherapy: ESMO clinical practice guidelines for diagnosis, treatment and follow-up. Ann Oncol. 2017;28(Suppl 4):iv119–42. 28881921 10.1093/annonc/mdx225

[6] Colle R Radzik A Cohen R. Pseudoprogression in patients treated with immune checkpoint inhibitors for microsatellite instability-high/mismatch repair-deficient metastatic colorectal cancer. Eur J Cancer. 2021;144:9–16. 33316636 10.1016/j.ejca.2020.11.009

[7] Sahin IH Akce M Alese O. Immune checkpoint inhibitors for the treatment of MSI-H/MMR-D colorectal cancer and a perspective on resistance mechanisms. Br J Cancer. 2019;121:809–18. 31607751 10.1038/s41416-019-0599-y PMC6889302

[8] Chae YK Wang S Nimeiri H Kalyan A Giles FJ. Pseudoprogression in microsatellite instability-high colorectal cancer during treatment with combination T cell mediated immunotherapy: a case report and literature review. Oncotarget. 2017;8:57889–97. 28915720 10.18632/oncotarget.18361 PMC5593692

[9] Parseghian CM Patnana M Bhosale P. Evaluating for pseudoprogression in colorectal and pancreatic tumors treated with immunotherapy. J Immunother. 2018;41:284–91. 29668571 10.1097/CJI.0000000000000222 PMC6028046

[10] Punt C Huiskens J van Gulik T. Pseudoprogression on bevacizumab treatment: tumor-dynamics in the modern era of systemic treatment for metastatic colorectal cancer. Acta Oncol. 2018;57:681–2. 29140135 10.1080/0284186X.2017.1374556

[11] Cohen R Bennouna J Meurisse A. RECIST and iRE ‐ CIST criteria for the evaluation of nivolumab plus ipilim ‐ umab in patients with microsatellite instability‐high/mis‐ match repair-deficient metastatic colorectal cancer: the GERCOR NIPICOL phase II study. J ImmunoTher Cancer. 2020;8:e001499. 33148693 10.1136/jitc-2020-001499 PMC7640587

[12] Colle R Radzik A Cohen R. Pseudoprogression in patients treated with immune checkpoint inhibitors for microsatellite instability-high/mismatch repair-deficient metastatic colorectal cancer. Eur J Cancer. 2021;144:9–16. 33316636 10.1016/j.ejca.2020.11.009

